# EET-Based Therapeutics Mitigate Sorafenib-Associated Glomerular Cell Damage

**DOI:** 10.3390/biom15091324

**Published:** 2025-09-16

**Authors:** Abhishek Mishra, Marcus de Bourg, Rawand S. Mohamed, Md Abdul Hye Khan, Tsigereda Weldemichael, Donald J. Johann, Samaneh Goorani, Shobanbabu Bommagani, Darin E. Jones, Anders Vik, John D. Imig

**Affiliations:** 1 Department of Pharmaceutical Sciences, University of Arkansas for Medical Sciences, Little Rock, AR 72205, USA; amishra@uams.edu (A.M.); makhan@uams.edu (M.A.H.K.); tgweldemichael@uams.edu (T.W.); sgoorani@uams.edu (S.G.); sbommagani@uams.edu (S.B.); dejones@uams.edu (D.E.J.); 2 Department of Pharmacy, Section for Pharmaceutical Chemistry, University of Oslo, 0316 Oslo, Norway; m.de.bourg@farmasi.uio.no (M.d.B.); m.rawand0404@gmail.com (R.S.M.); anders.vik@farmasi.uio.no (A.V.); 3 Department of Biomedical Informatics, University of Arkansas for Medical Sciences, Little Rock, AR 72205, USA; djjohann@uams.edu

**Keywords:** epoxylipids, onconephrology, mesangial cells, podocytes, nephrotoxicity

## Abstract

Background: This study investigates how sorafenib induces toxicity in glomerular cells and examines the protective role of 8,9-epoxyeicosatrienoic acid (8,9-EET) analogs in reducing this kidney damage. Methods: Human renal mesangial cells (HRMCs) and podocytes were treated with no treatment, sorafenib alone, or sorafenib combined with 8,9-EET analogs. Cell viability and apoptosis were measured in both cell types. Results: Sorafenib (1–10 µM) lowered cell viability and increased caspase 3/7 activity in a dose-dependent way in HRMCs and podocytes. Five of twenty 8,9-EET analogs significantly enhanced cell survival and decreased apoptosis. RNA sequencing showed that sorafenib altered 1244 genes, including those involved in cell cycle and the Raf/MEK/ERK pathway. The 8,9-EET analog MDB-52a raised ANGPTL4 levels, linked to metabolism and vascular health, and reduced ACTA2, which could activate protective pathways. Nephroseq data correlated these gene changes with glomerulosclerosis. Conclusions: MDB-52 appears to counteract gene disruptions and protect against sorafenib-induced kidney damage. Overall, 8,9-EET analogs targeting glomerular cells could be potential therapeutic agents to lessen sorafenib-related nephrotoxicity.

## 1. Introduction

Sorafenib and vascular endothelial growth factor tyrosine kinase inhibitors (VEGF-TKIs) are among the most commonly used treatment options for various cancers, especially renal and hepatocellular carcinoma [1,2]. The effectiveness of cancer chemotherapy using VEGF-TKIs, such as sorafenib, is significantly reduced by their nephrotoxicity [3,4]. This nephrotoxicity is crucial for patient prognosis, as glomerular injury is irreversible due to the limited recovery of essential renal filtration components [4]. Therefore, there is an urgent need for innovative strategies aimed at reducing the nephrotoxic effects of these drugs while maintaining their effectiveness in combating cancer.

Current strategies to reduce nephrotoxicity either slow down kidney function loss or offer no benefit, highlighting the urgent need for new solutions. We introduce a novel epoxylipid-based approach to lessen nephrotoxicity. Several strategies have been developed to maintain or increase EET bioavailability. One effective method that has shown promise in reducing kidney injury involves using synthetic mimetics or analogs of epoxylipids, called epoxyeicosatrienoic acid (EET). In previous studies, we demonstrated the beneficial effects of EET analogs in protecting the kidneys across various conditions, including cancer chemotherapy and radiotherapy-related nephrotoxicity [5,6,7]. Among the four EET isomers, analogs of 11,12-EET and 14,15-EET have been widely studied for their impacts on cardiovascular diseases, acute kidney injury, and chronic kidney disease, with positive outcomes [8,9]. Notably, analogs of 14,15-EET have shown kidney-protective effects against hypertension, renal fibrosis, renal allograft dysfunction [5,10,11,12,13], and chemotherapy-induced nephrotoxicity [14,15]. On the other hand, 8,9-EET and its analogs target the kidney specifically at the glomerulus [16]. Previous research indicates that 8,9-EET is the most common EET in the glomeruli and effectively lowers elevated glomerular permeability [16]. In this context, we suggest that the distinct glomerular action of 8,9-EET analogs could be beneficial in treating VEGF-TKI-induced renal injury, which primarily involves glomerular damage [15]. We synthesized a series of synthetic mimetics of 8,9-EET and recently demonstrated important biological actions against sorafenib nephrotoxicity in human renal mesangial cells [17]. In the present study, we have conducted an in-depth study involving two major glomerular cell types to determine the renal cytoprotective actions of these 8,9-EET analogs. Glomerular cell RNA sequencing and bioinformatics analysis of transcriptomic data provided insights into the molecular mechanisms underlying VEGFi-induced renal cytotoxicity. Transcriptomic data analysis identified five novel 8,9-EET analogs that demonstrated promising effects against sorafenib-induced glomerular cytotoxicity and established a lead analog for further development.

## 2. Materials and Methods

### 2.1. Chemicals

Sorafenib was purchased from LC Laboratories (Broadview Heights, OH, USA). The caspase 3/7 dye was obtained from Sartorius (Bohemia, NY, USA). Cell viability WST assays were performed using Cell Counting Kit 8 from Abcam (Cambridge, MA, USA). All reagents for real-time PCR (RT-qPCR) were acquired from Thermo Fisher Scientific (Waltham, MA, USA). Analogs of 8,9 EET were sourced from the University of Oslo (Oslo, Norway). A list of the analogs used in this study is provided in Table 1.

### 2.2. Methodology

Human renal mesangial cells (HRMCs; ScienCell, Carlsbad, CA, USA) were cultured in RPMI 1640 medium (Gibco, NY, USA) supplemented with 10% FBS, 100 U/mL penicillin, and 0.1 mg/mL streptomycin at 37 °C in a 5% CO_2_ environment. Human podocytes, obtained from the University of Bristol (Bristol, UK), were conditionally immortalized and initially cultured in RPMI 1640 medium (Gibco, Grand Island, NY, USA) supplemented with 10% fetal bovine serum (FBS), 100 U/mL penicillin, and 0.1 mg/mL streptomycin. Cultures were maintained at 33 °C in a 5% CO_2_ atmosphere to promote cellular proliferation. Once the cells reached 35–50% confluence, the incubation temperature was increased to 37 °C—a process referred to as “thermo-switching”—to initiate differentiation. Following the temperature shift, the culture medium was replaced with RPMI 1640 supplemented with 1% FBS, 100 U/mL penicillin, and 0.1 mg/mL streptomycin. Cells were then maintained at 37 °C in a 5% CO_2_ environment. Within 24 h, confluence increased to approximately 70–80%. The cultures were subsequently maintained at 37 °C for an additional 2–3 weeks to allow for full maturation. Maturation was confirmed via RT-qPCR analysis of podocyte-specific markers, including podocin and nephrin. This protocol was adapted from previously published methods [18,19] with modifications including a lower initial confluence and reduced serum concentration during the maturation phase to promote differentiation while minimizing further proliferation.

Human prostate cancer cells (DU145, Cat. # ATCC-HTB-81; Manassas, VA, USA) were cultured at 37 °C in a 5% CO_2_ atmosphere using Dulbecco’s Modified Eagle Medium (DMEM, Cat. # ATCC-30-2006; Manassas, VA, USA), supplemented with 100 U/mL penicillin and 0.1 mg/mL streptomycin. Cells were subcultured following the manufacturer’s protocol.

### 2.3. Cell Viability Assay

The effect of sorafenib on the HMRC viability and podocytes was determined in a 96-well plate by exposing 24 h serum-starved cells to one of the following treatments: control (no treatment) or sorafenib (1, 3, 5, or 10 µM). In a separate protocol, the protective effects of 8,9-EET analogs on the viability of sorafenib-treated HRMCs and podocytes were assessed by exposing serum-starved cells to sorafenib alone or to sorafenib plus 8,9-EET analogs (1, 3, or 10 µM). The doses of sorafenib used on HRMCs were 5 and 10 µM, while podocytes were treated only with 10 µM. In both protocols, after 48 h of incubation, a WST-1 assay was employed to assess cell survival. Ten microliters of WST reagent were added to each well, followed by a 2 h incubation at 37 °C. Cellular dehydrogenases reduce the WST-8 tetrazolium salt in the assay medium to an orange formazan product, which is soluble in the tissue culture medium. The amount of formazan produced, measured as absorbance at 460 nm, is directly proportional to the number of viable cells. The absorbance at 460 nm was determined using a BioTek Synergy 4 Microplate Reader (Agilent Technologies, Santa Clara, CA, USA).

### 2.4. Caspase 3/7 Activation for Apoptosis Analysis

Caspase 3/7 activation in human renal mesangial cells (HRMCs) and podocytes was assessed using the IncuCyte^®^ SX5 live-cell analysis system (Sartorius, Göttingen, Germa-ny) over 48 h. Cells were seeded at a density of 5000 cells per well in 96-well TPP plates to facilitate optimal adherence. After attachment, cells were incubated with Caspase 3/7 dye (prepared in RPMI 1640 medium) and subjected to one of five treatment conditions: (1) untreated control, (2) sorafenib (10 µM), or sorafenib (10 µM) in combination with an 8,9-epoxyeicosatrienoic acid (8,9-EET) analog at concentrations of 1 µM, 3 µM, or 10 µM. Each 96-well plate was designed to test two to three compounds, including matched vehicle and sorafenib (10 µM) controls. The same control datasets were used for analyzing caspase 3/7 activation across both compounds tested on each plate. This standardized protocol was consistently applied across all experiments involving HRMCs and podocytes. Real-time imaging was performed using the 10× objective of the IncuCyte^®^ system, with image acquisition every 3 h for 48 h. Both green fluorescence (to detect caspase 3/7 activation) and phase contrast channels were utilized to monitor cell morphology and apoptotic activity.

### 2.5. Cell Confluence Analysis

Cell confluence in HRMCs and podocytes was evaluated using Incucyte live-cell imaging and its corresponding software program.

### 2.6. Genomic Study

#### 2.6.1. RNA-Seq Data Analysis of HRMCs

Treated HRMCs underwent processing for RNA extraction using a Quick-DNA/RNA FFPE Miniprep Kit, which featured on-column DNase digestion for RNA preparations (Zymo Research, Irvine, CA, USA). The RNA was evaluated for mass concentration with the Qubit RNA Broad Range Assay Kit (Invitrogen, Carlsbad, CA, USA) utilizing a Qubit 4 fluorometer (Invitrogen, Carlsbad, CA, USA). RNA quality was assessed using a Standard Sensitivity RNA Analysis Kit (Agilent, Santa Clara, CA, USA) on a Fragment Analyzer System (Agilent, Santa Clara, CA, USA). Sequencing libraries were created with the TruSeq Stranded Total RNA Library Prep Gold (Illumina, San Diego, CA, USA). RNA DV200 scores determined fragmentation times. Libraries were evaluated for mass concentration using a Qubit 1× dsDNA HS Assay Kit (Invitrogen, Carlsbad, CA, USA) in conjunction with a Qubit 4 fluorometer. Library fragment size was analyzed using a High Sensitivity NGS Fragment Analysis Kit (Agilent, Santa Clara, CA, USA) on a Fragment Analyzer System. Libraries were functionally validated using a KAPA Universal Library Quantification Kit (Roche, Indianapolis, IN, USA). Sequencing was performed to generate paired-end reads (2 × 100 bp) using a 200-cycle S1 flow cell on an Illumina NovaSeq 6000 sequencing system(Illumina, San Diego, CA, USA).

#### 2.6.2. Bioinformatics Analysis

We examined the mRNA expression profiles of three replicate samples from the five HRMC groups. The samples were sequenced on an NGS platform. The files containing the sequencing reads (FASTQ) were tested for quality control using MultiQC. The Cutadapt tool was employed to trim the Illumina adapter and low-quality bases. After quality control, the reads were aligned to a reference genome (mm10/GRCm38) using the HISAT2 aligner [20], followed by mapping the counting reads to RefSeq genes with feature counts. We generated the count matrix from the sequence reads using HTSeq-count [21]. Genes with low counts across the samples impact the false discovery rate, thereby reducing the power to detect differentially expressed genes; thus, before identifying differentially expressed genes, we filtered out genes with low expression using a module in the limma-voom tool [22]. Next, we normalized the counts using TMM normalization, as reported earlier by Robinson and Oshlack [23], a weighted trimmed mean of the log expression proportions employed to scale the counts of the samples. Differentially expressed genes (DRGs) were defined using a threshold of |log2FC| ≥ 1 and adjusted *p*-value < 0.05. Finally, a linear model was fitted using limma to identify DRGs, with data expressed as mean ± standard error of the mean. All *p*-values were adjusted for multiple comparisons employing the Benjamini–Hochberg False Discovery Rate (FDR) correction. A complete list of the genes involved in identifying DRGs is provided in Supplementary Tables S2–S4.

#### 2.6.3. RT-qPCR

RT-qPCR validated the RNA-seq results of selected genes. RNA was extracted from sample homogenates using a RNeasy Mini Kit (Qiagen, Germantown, MD, USA) following the manufacturer’s protocol. Spectrophotometric quantification of RNA samples was performed using a NanoDrop (Thermo Scientific, Waltham, WA, USA, NanoDrop One). Subsequently, equal amounts of RNA were reverse transcribed with an RT2 Easy First Strand Kit for cDNA synthesis (Applied Biosystems, Waltham, MA, USA), diluting them in nuclease-free water to achieve a final concentration of 5 ng/μL. β-actin served as a housekeeping gene for value normalization. At the same time, target detection and quantification were conducted using SYBR Green chemistry (Applied Biosystems) on an ABI QuantStudio 6 Pro real-time PCR system. Samples were denatured at 95 °C for 2 min. Next, PCR was performed on triplicate samples using a protocol of 40 cycles at 95 °C for 10 s and at 60 °C for 30 s. Relative expression was calculated using the ΔΔCt method. Statistical analyses were performed using five samples from each experimental group and were compared to the control group.

### 2.7. Statistical Analysis

Quantitative data are presented as mean ± standard error of the mean (SEM). Statistical analyses were conducted using GraphPad Prism^®^ Version 9.0. For comparisons involving multiple groups, one-way ANOVA followed by Tukey’s multiple comparisons test was used. For direct comparisons between two groups, two-tailed unpaired Student’s *t*-tests were performed. A *p*-value of ≤0.05 was considered statistically significant. For transcriptomic analysis, raw sequencing reads were processed and aligned to the GRCh37/hg19 reference genome using HISAT2, and gene-level counts were obtained with HTSeq-count. Genes with low expression levels were filtered out, and normalization was performed using the trimmed mean of M-values (TMM) method. Differential gene expression was analyzed using the limma-voom pipeline, with *p*-values adjusted for multiple testing through the Benjamini–Hochberg false discovery rate (FDR) correction. Genes with an absolute log2 fold change ≥ 1 and FDR-adjusted *p* < 0.05 were deemed significantly differentially expressed.

## 3. Results

### 3.1. Sorafenib Treatment Decreases Cell Viability

**Figure 1:**
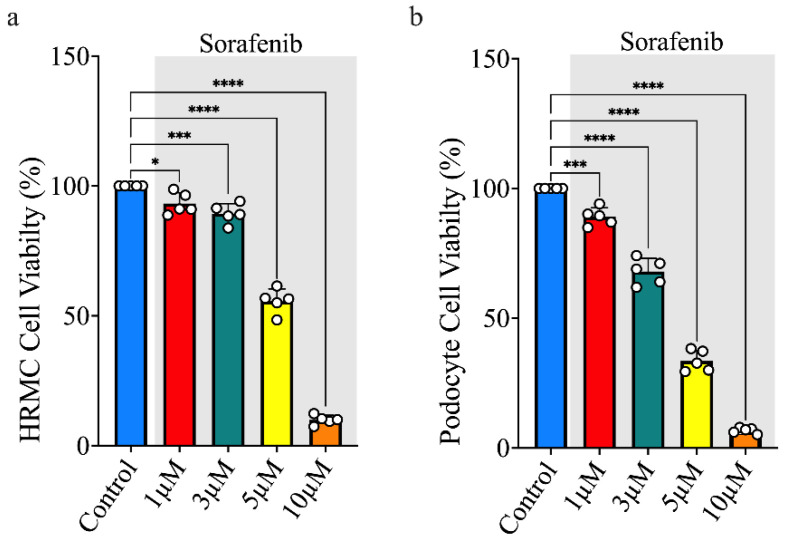
Sorafenib treatment reduces cell viability in a dose-dependent manner. (**a**) Cell viability measured in HRMCs treated with increasing concentrations of sorafenib (1, 3, 5 and 10 µM). The data demonstrate a dose-dependent reduction in cell viability, indicating the cytotoxic effects of sorafenib on this cell. (**b**) Cell viability measured in podocyte cells treated with serial dilutions of sorafenib. Similarly to HRMCs, podocytes show a dose-dependent decrease in viability, suggesting sensitivity to sorafenib-induced cytotoxicity across different glomerular cell types. These results highlight the dose-dependent impact of sorafenib on glomerular cell viability. White dots indicate individual samples; * *p* < 0.05, *** *p* < 0.01, and **** *p* < 0.001 indicates statistical significance.

HRMCs and podocytes were exposed to either no treatment (controls) or varying concentrations of sorafenib (1, 3, 5, or 10 µM) and were assessed for their viability. Cells treated with sorafenib exhibited a dose-dependent decrease in cell viability. HRMCs exposed to 1 or 3 μM sorafenib had a mild effect on cell viability, whereas those treated with 5 μM exhibited a 45–55% reduction in cell viability compared to the control (Figure 1a). Furthermore, HRMCs treated with 10 μM sorafenib demonstrated significantly reduced cell viability, with 88–95% fewer cells compared to the control group. In podocytes, we observed a 90–92% reduction in cell viability with 10 μM sorafenib, a 30% reduction with 3 μM, and a 60% reduction with 5 μM sorafenib, relative to the control. Similarly to HRMCs, podocytes treated with 1 μM sorafenib showed no impact on cell viability (Figure 1b).

### 3.2. Synthetic Analogs of 8,9-EET Maintained the Viability of HRMCs Treated with Sorafenib

**Figure 2:**
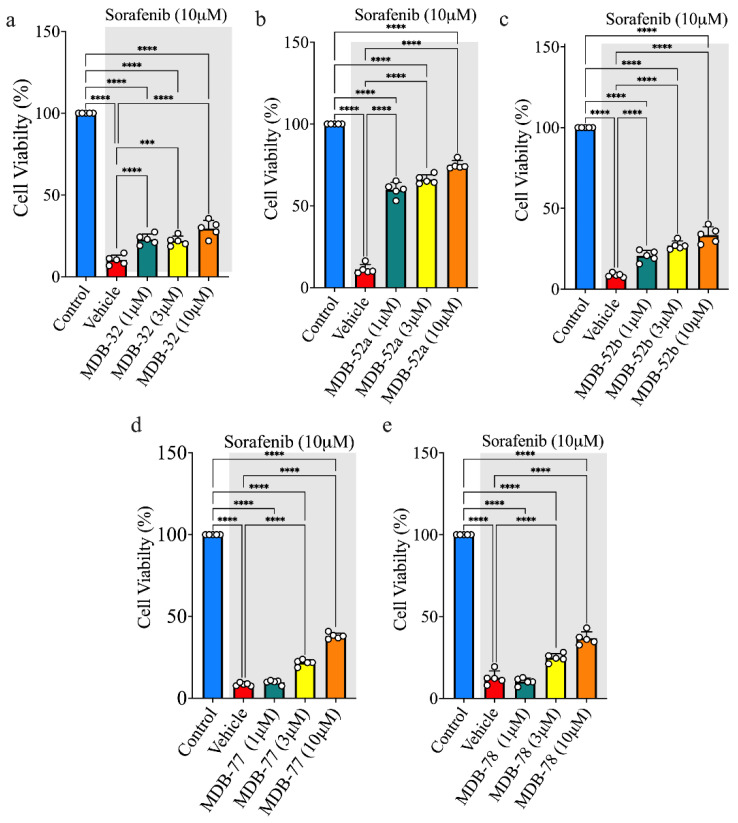
Protective effects of an 8,9-EET analog on sorafenib treatment in preserving HRMC viability in a dose-dependent manner. (**a**) MDB-32 significantly increases cell viability, providing protection against sorafenib-induced cell death in HRMCs. (**b**) MDB-52a shows improved cell viability across all tested concentrations (1, 3, and 10 µM), with the 10 µM dose nearly restoring cell viability to the levels observed in control cells. (**c**–**e**) MDB-52b, MDB-77, and MDB-78 exhibit a dose-dependent increase in cell viability, with higher concentrations leading to greater protection against sorafenib-induced cytotoxicity. These results highlight the potential of these 8,9-EET analogs in enhancing cell survival under sorafenib treatment, with MDB-52a demonstrating the most significant effect at higher doses. White dots indicate individual samples; *** *p* < 0.01, and **** *p* < 0.001 indicates statistical significance.

We evaluated the protective effects of twenty 8,9-EET analogs against sorafenib-induced glomerular cell toxicity in HRMCs exposed to 5 µM sorafenib (Supplementary Figure S1). Among these, five analogs, MDB-52a, MDB-52b, MDB-58, MDB-77, and MDB-78, demonstrated significant cytoprotective effects (Supplementary Figure S1g,h,i,k,l). The remaining analogs showed little to no protective activity at this concentration. Next, we tested the ability of these analogs to improve HRMC viability at a higher sorafenib concentration (10 µM). Consistent with earlier results, fifteen analogs failed to restore cell viability above the 25% threshold, indicating limited effectiveness (Supplementary Figure S2). However, MDB-32, MDB-52a, MDB-52b, MDB-77, and MDB-78 exhibited strong protective effects, with MDB-52a providing the most significant cytoprotection against 10 µM sorafenib-induced HRMC death (Figure 2b).

### 3.3. Synthetic Analogs of 8,9-EET Mitigate Sorafenib-Induced HRMCs Apoptosis

**Figure 3:**
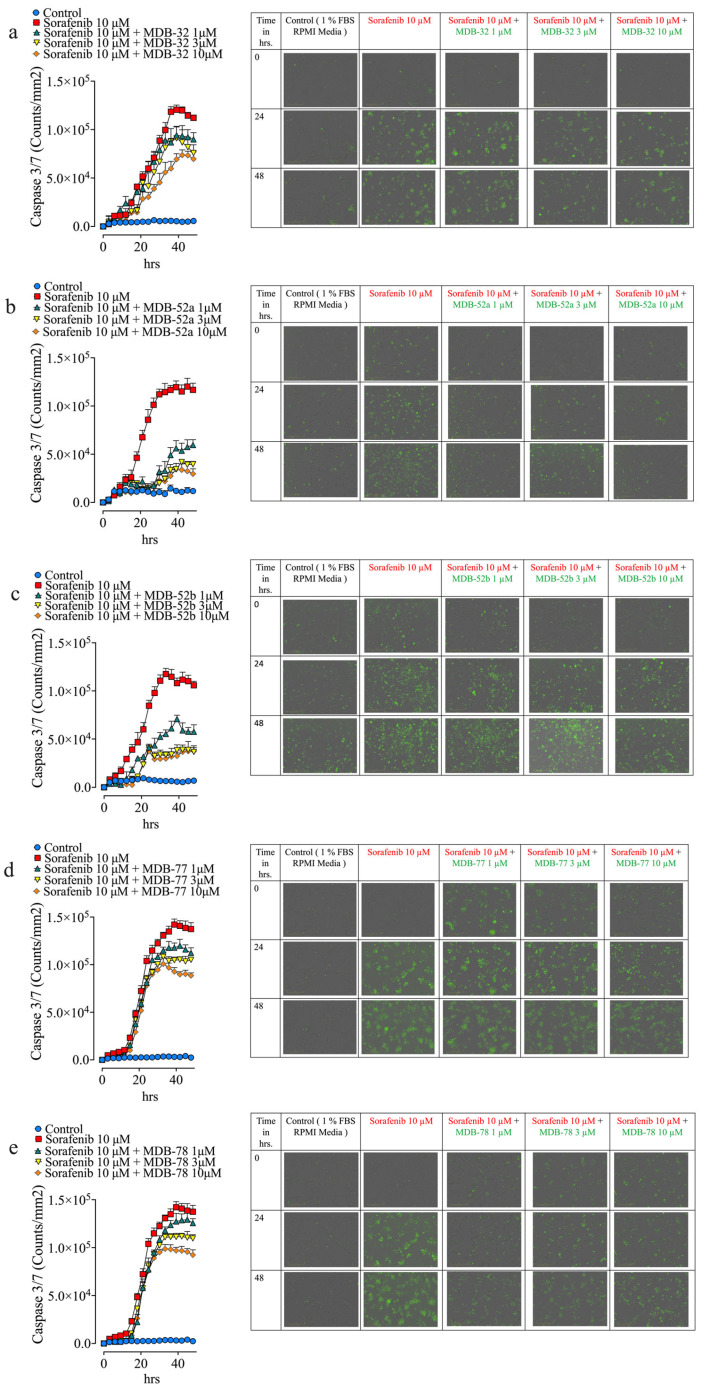
8,9-EET Analog Mitigates Sorafenib-Induced HRMCs Death by Suppressing Caspase 3/7 Activity. Caspase 3/7 activity, a marker of sorafenib-induced apoptosis, is visualized as green, fluorescent spots. An increase in green fluorescence indicates elevated caspase 3/7 activity and greater cell death, while a decrease reflects reduced apoptotic activity. HRMCs were seeded into 96-well plates for compound screening. Each plate was used to test two to three compounds, alongside matched vehicle and sorafenib (10 µM) controls. Caspase 3/7 activation was assessed using a luminescent assay, with control datasets applied uniformly across all compounds tested on the same plate. MDB-32, MDB-52a, and MDB-52b were each tested on separate 96-well plates, with individual sets of vehicle and sorafenib controls specific to each compound. In contrast, MDB-77 and MDB-78 were tested concurrently on a single 96-well plate, sharing a common set of vehicle and sorafenib controls. This design enabled consistent intraplate comparisons while maintaining compound-specific control conditions. (**a**) Treatment with the 8,9-EET analog MDB-32 led to a 20–40% reduction in sorafenib-induced caspase 3/7 activity, suggesting a protective effect against apoptosis. (**b**) Cells treated with MDB-52a in combination with 10 µM sorafenib showed significantly lower caspase 3/7 activity compared to sorafenib alone. MDB-52a reduced activity in a dose-dependent manner by 60–90%, demonstrating strong anti-apoptotic efficacy in HRMC cells. (**c**) A similar reduction in caspase 3/7 activity was observed with MDB-52b, further supporting its protective role. (**d**,**e**) HRMC cells treated with MDB-77 and MDB-78 also exhibited decreased caspase 3/7 activity relative to sorafenib-treated controls, indicating potential anti-apoptotic effects of these compounds.

Live cell imaging demonstrated that sorafenib caused increased HRMC caspase 3/7 activity. Five analogs (MDB-52a, MDB-52b, MDB-58, MDB-77, and MDB-78) significantly reduced sorafenib-induced HRMC caspase 3/7 activity. Among these five, MBD-32 treatment decreased sorafenib-induced caspase 3/7 activity by 20–45% in sorafenib-treated HRMCs, while MDB-77 and MDB-78 lowered caspase 3/7 activity in a dose-dependent manner by up to 50% (Figure 3a-e. Interestingly, MDB-52a and MDB-52b treatment diminished caspase 3/7 activity in a dose-dependent manner by up to 90%. MDB-52a exhibited superior dose–response cytoprotective effects, safeguarding HRMCs from sorafenib-induced apoptosis at 1, 3, or 10 µM MDB-52a (Figure 3b,c). Cell confluence also indicated that MDB-52a offers enhanced protection against sorafenib-induced cytotoxicity in HRMCs (Supplementary Figure S4b). These findings illustrate that 8,9-EET analogs shield HRMCs from sorafenib nephrotoxicity by preventing caspase 3/7-dependent apoptotic cell death.

### 3.4. Synthetic Analogs of 8,9-EET Maintain Podocyte Cell Viability During Sorafenib Exposure

**Figure 4:**
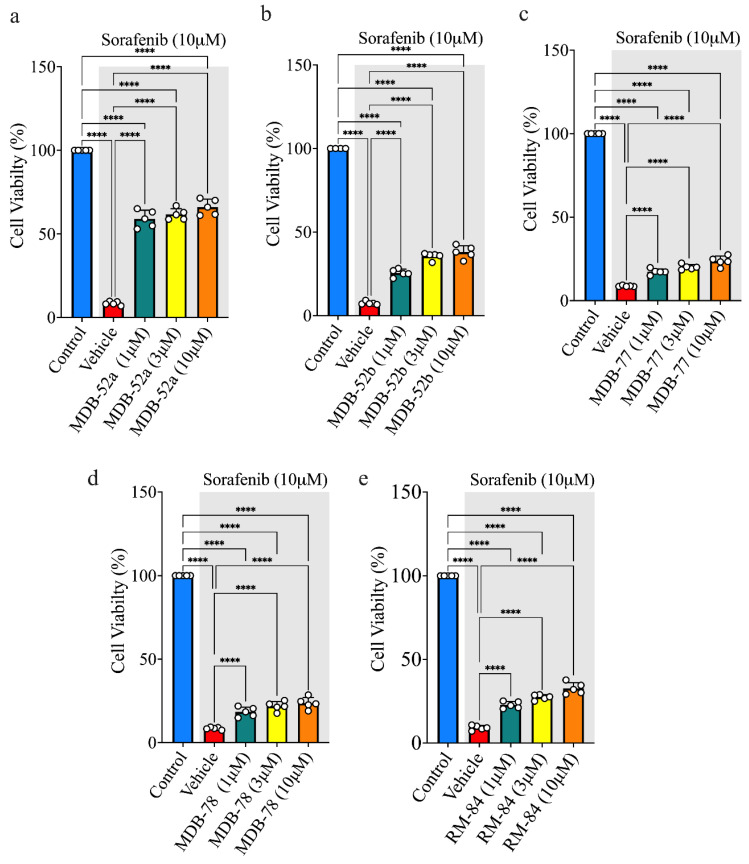
Protective effects of an 8,9-EET analog on sorafenib treatment in preserving podocyte cell viability in a dose-dependent manner. (**a**) MDB-52a significantly change in cell viability in podocyte cells, providing protection against sorafenib-induced cell death, with the 10 µM dose showing the most notable improvement. (**b**) MDB-52b enhances cell viability across all tested concentrations (1, 3, and 10 µM), with the 10 µM dose nearly restoring cell viability to 50%. (**c**–**e**) MDB-77, MDB-78, and RM-84 exhibit a dose-dependent increase in cell viability, with higher concentrations offering greater protection against sorafenib-induced cytotoxicity. These findings underscore the potential of 8,9-EET analogs in protecting cell survival during sorafenib treatment, with MDB-52a showing the most pronounced effect at 10 µM in podocyte cells. White dots indicate individual samples; **** *p* < 0.001 indicates statistical significance.

We also investigated the effects of novel 8,9-EET analogs on the viability of podocytes exposed to 10 µM sorafenib using five treatments: control (no treatment), sorafenib (10 µM), and sorafenib (10 µM) plus 8,9-EET analogs (1, 3, or 10 µM). As observed in HRMCs, the five 8,9-EET analogs exhibited protective effects on podocytes against sorafenib-induced nephrotoxicity (Supplementary Figure S3). Podocytes treated with 10 µM sorafenib showed pronounced cytotoxicity, with a cell viability range of 7–11% compared to the control (Figure 1b). Five 8,9-EET analogs, MDB-52a, MDB-52b, MDB-77, MDB-78, and RM-84, demonstrated strong protective effects against sorafenib cytotoxicity in podocytes (Figure 4). Among these, MDB-52a conferred the most significant protection against sorafenib compared to the other compounds.

### 3.5. Synthetic 8,9-EET Analogs Reduce Apoptosis Caused by Sorafenib

**Figure 5:**
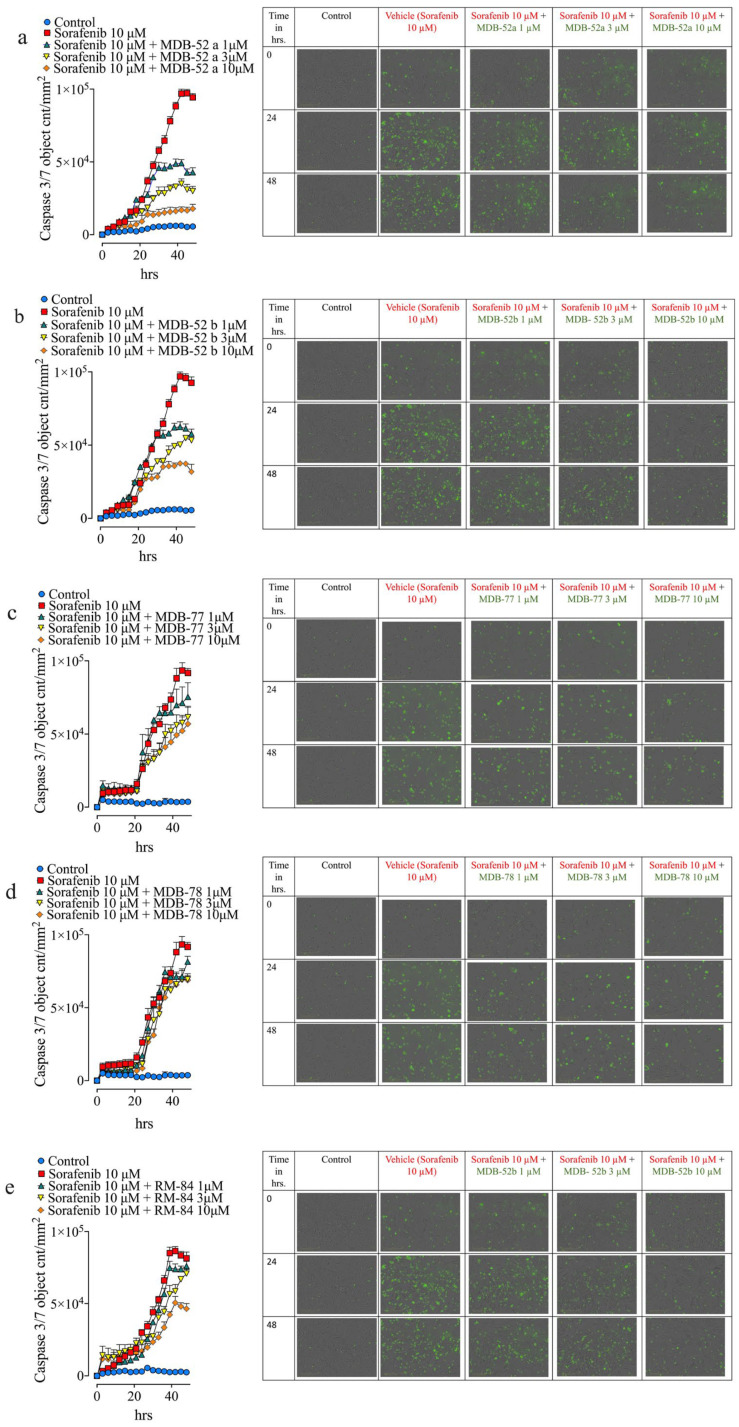
8,9 EET Analog Mitigates Sorafenib-Induced Cell Death of Podocyte by Suppressing Caspase 3/7 Activity. The protective potential of 8,9-EET analogs against sorafenib-induced apoptosis in podo-cytes was evaluated by measuring caspase 3/7 activity, a key marker of programmed cell death. Apoptotic activity was visualized as green, fluorescent spots, an increased number of spots indi-cates elevated caspase 3/7 activity, while fewer spots suggest reduced apoptosis. Human podo-cytes were seeded into 96-well plates to evaluate caspase 3/7 activation in response to various test compounds. Each plate was configured to include two to three compounds, along with matched vehicle and sorafenib (10 µM) controls. A single set of control data (vehicle and sorafenib) was used for all compounds tested on the same plate to ensure consistent intra-plate comparisons. MDB-52a, MDB-52b, and RM-84 were tested concurrently on one 96-well plate, sharing a common set of vehicle and sorafenib controls. Similarly, MDB-77 and MDB-78 were tested together on a separate plate, also using a single set of vehicle and sorafenib controls for both compounds. Treatment with 8,9-EET analogs significantly reduced caspase 3/7 activity induced by sorafenib, indicating their protective effects: (**a**) MDB-52a: Co-treatment with MDB-52a and 10 µM sorafenib resulted in a marked reduction in caspase 3/7 activity compared to sorafenib alone. The effect was dose-dependent, with a reduction of approximately 50–70%. (**b**) MDB-52b: Similar protective effects were observed, with caspase 3/7 activity reduced by 40–60% in a dose-dependent manner. (**c**,**d**) MDB-77 and MDB-78: Both compounds decreased caspase 3/7 activity when combined with sorafenib. MDB-77 was effective at 1 and 3 µM, while MDB-78 showed minimal effect at lower doses but was effective at 10 µM. (**e**) RM-84: This analog also demonstrated a dose-dependent reduction in caspase 3/7 activity, with the 10 µM dose significantly lowering apoptosis compared to sorafenib-only treatment.

Podocytes treated with sorafenib and 1 µM MDB-52a exhibited a 55% reduction in apoptosis, whereas apoptosis decreased by 70% at the 3 µM and by 85% at the 10 µM (Figure 5). The combined data from the apoptosis, cell viability, and confluence assays indicated that MDB-52a is the most effective at protecting kidney cells from the adverse effects of sorafenib among all twenty 8,9-EET analogs evaluated in this study (Figure 4 and Figure 5, and Supplementary Figure S5a).

### 3.6. Synthetic Analog of 8,9-EET, MDB-52a, Does Not Interfere with Sorafenib’s Anticancer Activity

**Figure 6:**
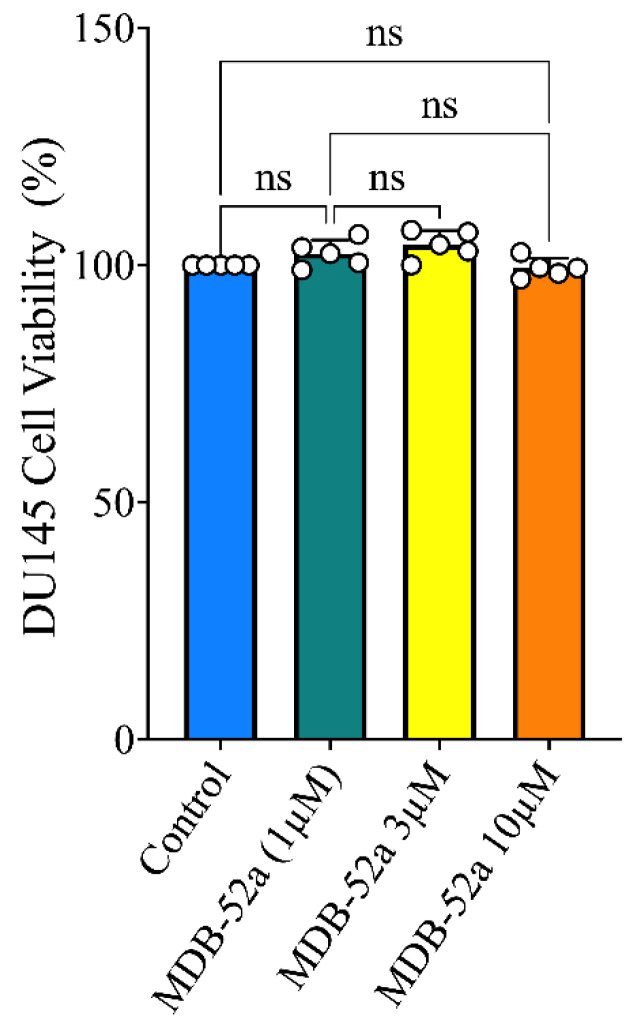
Evaluation of the 8,9 EET analog mdb-52a for anticancer activity and tumor growth promotion. Human prostate cancer cells treated with MDB52a (1, 3 & 10 µM) showed no significant antitumor effects, indicating that MDB52a lacks inherent anticancer properties. Additionally, MDB52a did not promote tumor growth, suggesting it is a neutral compound in terms of cancer cell proliferation within this concentration range.

We assessed whether MDB-52a exhibits any intrinsic antitumor activity. To explore this, human prostate cancer cells were treated with varying concentrations of MDB-52a, from 1 to 10 μM (Figure 6). The results indicated no significant antitumor effects within this concentration range, suggesting that MDB-52a does not inherently possess anticancer properties and does not promote tumor growth. This finding is important because it implies that introducing MDB-52a would not compromise the anticancer efficacy of sorafenib.

### 3.7. Sorafenib and Synthetic Analog MDB52a Affect Gene Expression in HRMC

The RNA sequencing study on MDB-52a provided insights into the mechanism of action at the signaling pathway level. In this analysis, we used five experimental groups: Group A (HMRC control), Group B (HMRCs treated with 10 µM sorafenib), Group C (HMRCs treated with 10 µM sorafenib and 3 µM MDB-52a), Group D (HMRCs treated with sorafenib and 10 µM MDB-52a), and Group E (HMRCs treated with 10 µM MDB-52a). Comparisons between untreated cells (Group A) and those treated with sorafenib (Group B) showed that 1244 genes were significantly differentially expressed. Examining the top 50 significantly differentially expressed genes, we found that key genes involved in the cell cycle pathway, such as CDC25, E2 F2, MCM10, and MYBL2, were downregulated, while ANKK1, DUSP9, CYP27B1, and RRAGD were upregulated. When comparing Group B (sorafenib) with Groups A (control) and D (sorafenib and MDB-52a), we observed that RP11-178L8.4 was downregulated in sorafenib and upregulated in sorafenib with MDB-52a.

**Figure 7:**
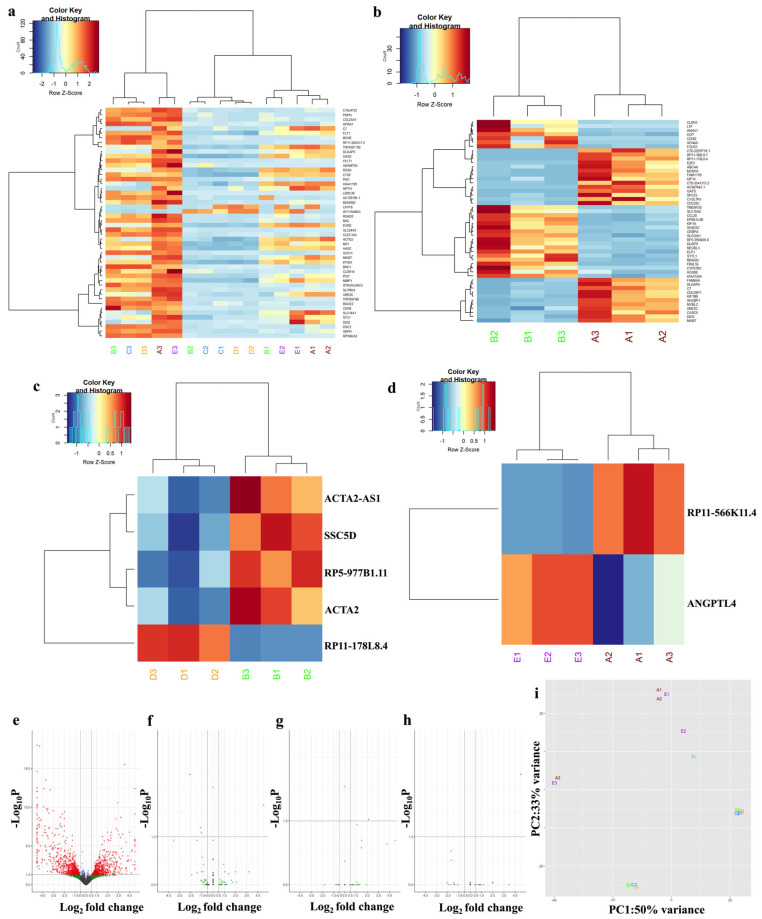
RNA-Seq analysis identifies key differentially expressed genes (DEGs) in HRMCs treated with 8,9-EET analog mdb-52a. This figure presents RNA sequencing (RNA-seq) analysis to identify key DEGs in HRMCs treated with 8,9-EET analogs. The volcano plot highlights the distribution of genes based on statistical significance. Red points represent genes that are statistically significant, meeting the threshold for both *p*-value (<0.05) and fold change (log2FC). The position along the *x*-axis indicates whether a gene is upregulated or downregulated. Red points on the right (positive log2 fold change) represent upregulated genes (i.e., genes expressed more in the experimental condition), while red points on the left (negative log2 fold change) represent downregulated genes (i.e., genes expressed less in the experimental condition). Green points represent non-significant genes: those on the left are non-significant downregulated genes, while those on the right are non-significant upregulated genes. While these genes meet the fold change threshold (either up or down), they fail to meet the significance threshold based on *p*-value (padj ≥ 0.05). This analysis provides an overview of how MDB-52a affect gene expression in HRMCs. (**a**) Heat map displaying the 50 most significantly differentially expressed genes across Groups A–E. (**b**) A DEG expression heatmap displaying the 50 most upregulated and downregulated genes between Groups A and B. These results highlight the extensive gene expression changes between the untreated group and the treatment group. (**c**) Heat map of Group D vs. Group B shows five significantly differentially expressed genes. These results indicate significant gene expression changes between the two groups, highlighting the impact of treatment with sorafenib alone compared to the combined treatment of sorafenib with MDB52a. (**d**) Heat map of Group E vs. Group A shows two significantly differentially expressed genes. These figures indicate significant gene expression changes in HRMCs treated with MDB52a compared to untreated cells. (**e**) The volcano plot comparing Group A and Group B shows that a total of 1244 genes were significantly differentially expressed. (**f**) Volcano plot displaying mRNA expression differences between Group D and Group B (**g**) Volcano plot displaying mRNA expression differences between Group E and Group A (**h**) Volcano plot displaying mRNA expression differences between Group C and Group B (**i**) Analysis of 1000 differentially expressed genes using a PCA plot revealed distinct clustering patterns. Groups B, C, and D exhibited similar gene expression levels, indicating related responses to their respective treatments. In contrast, Groups A (untreated) and E (treated with MDB52a) displayed similar gene reactivation patterns, suggesting that MDB52a may restore normal gene expression, mimicking the untreated condition. Group-A, without any treatment, Group-B, cells treated with 10 µM Sorafenib, Group-C (cells treated with 3 µM MDB-52a and 10 µM Sorafenib) Group-D (cells treated with 10 µM MDB-52a and Sorafenib) and Group-E (cells treated with 10 µM MDB-52a (8,9 EET analog).

Additionally, genes ACTA-2, SSC-5D, RP5-977B1.11, and ACTA2-AS1 were notably downregulated in Group D (sorafenib and MDB-52a) compared to Group B (sorafenib). These results indicate that the 8,9-EET analog MDB-52a affects gene regulation in HRMCs. Figure 7 further supports this activation. Notably, ANGPTL4 and RP11-566K11.4 were highly expressed in Group E, treated with the 8,9-EET analog alone. PCA analysis showed distinct clustering, with Groups B (sorafenib), C (sorafenib plus 3 µM MDB-52a), and D (sorafenib plus 10 µM MDB-52a) displaying similar gene expression profiles, indicating a common response. Conversely, Groups A (untreated) and E (MDB-52a) exhibited similar activation patterns, suggesting a shared underlying gene activation mechanism (Figure 7). RT-qPCR validated the RNA-seq data for selected genes.

### 3.8. MDB-52a Influences Sorafenib-Induced Alterations in Podocyte-Specific Gene Expression

**Figure 8:**
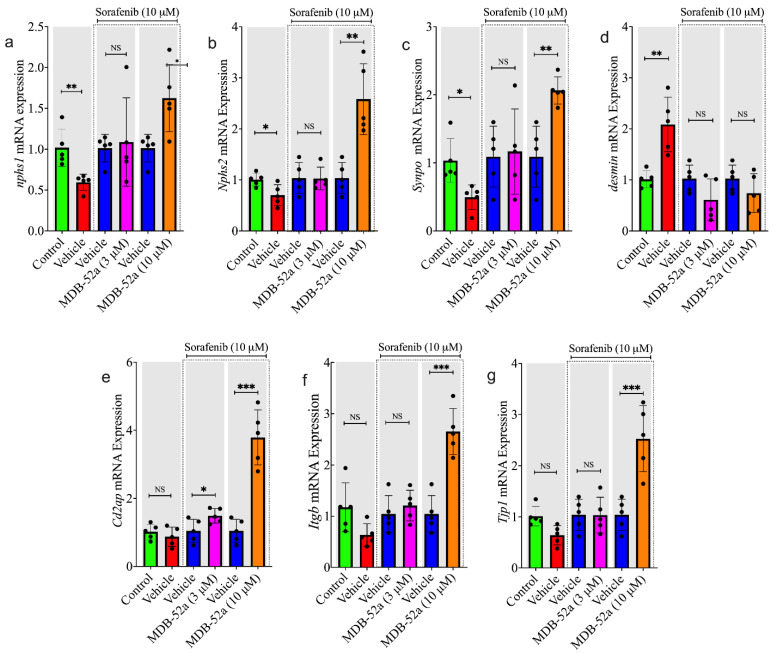
MDB-52a effects on sorafenib-induced changes in podocyte-specific gene expression. Relative mRNA expression levels of key podocyte markers, including (**a**) NPHS1, (**b**) Desmin, (**c**) Synaptopodin, (**d**) NPHS2, (**e**) CD2AP, (**f**) ITGB, and (**g**) TJP1, were quantified using RT-PCR across different experimental groups. Group A represents untreated control cells with baseline expression levels. Group B (10 µM Sorafenib-treated cells) exhibited a significant reduction in protective markers (NPHS1, NPHS2, Synaptopodin, TJP1, CD2AP, ITGB) and a marked increase in the injury marker Desmin, indicating podocyte injury. Co-treatment with a low concentration of MDB-52a (Group C: 3 µM MDB-52a + 10 µM Sorafenib) showed minor improvements in some markers but was insufficient for substantial recovery. However, co-treatment with a higher concentration of MDB-52I (Group D: 10 µM MDB-52a + Sorafenib) significantly restored the expression of protective markers to near-normal levels and mitigated the upregulation of Desmin, highlighting the robust protective effects of MDB-52a at higher doses. Data are presented as mean ± SEM, and statistical significance was determined using one-way ANOVA with Tukey’s post hoc test (* *p* < 0.05, ** *p* < 0.01, *** *p* < 0.001, NS: not significant).

Gene expression of podocyte-specific proteins NPHS1, NPHS2, synaptopodin, CD2AP, ITGB, and TJP1 was significantly reduced in cells treated with sorafenib compared to the control group. Meanwhile, in sorafenib-treated podocytes, there was a notable increase in the injury marker desmin, highlighting the damaging effect of sorafenib on podocytes. Co-treatment with 3 µM MDB-52a showed improvement in gene expression related to podocyte structure and function. However, these changes neither fully restored gene expression nor significantly decreased podocyte injury markers (Figure 8). In contrast, co-treatment of sorafenib-treated podocytes with a higher dose of MDB-52a at 10 µM significantly affected the expression of protective podocyte markers like NPHS1, synaptopodin, and TJP1, bringing their gene levels close to normal and significantly reducing desmin expression. These findings confirm the glomerular protective effects of MDB-52a against sorafenib-induced cytotoxicity.

### 3.9. Naphroseq Analysis Identifies Genes Influenced by MDB-52a as Therapeutic Targets

**Figure 9:**
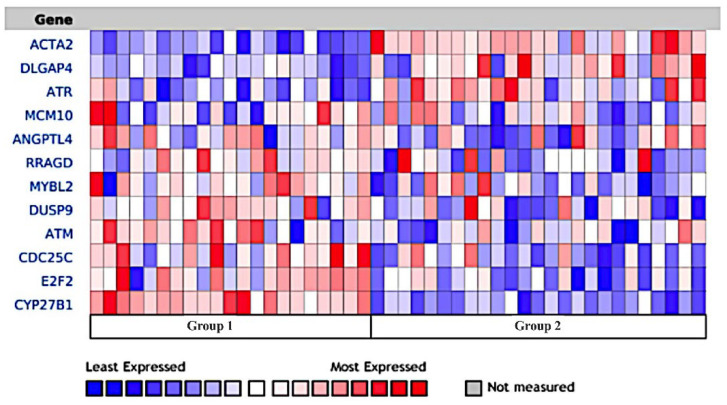
Comparative expression analysis of selected genes in focal segmental glomerulosclerosis (FSGS) vs. healthy living donors. This figure presents a comparison of gene expression between patients with Focal Segmental Glomerulosclerosis (FSGS) (Group 2) and Healthy Living Donors (Group 1) using log2 median-centered intensity. The heatmap displays gene expression levels, with red indicating higher expression and blue indicating lower expression. The analysis highlights significant differences in gene expression between the two groups, helping to illustrate key molecular changes associated with FSGS. Data were obtained and visualized using Nephroseq (The Regents of The University of Michigan, Ann Arbor, MI, USA). For additional details, the Nephroseq resource can be accessed at: http://www.nephroseq.org/resource/main.html (accessed on 15 February 2024). Group 1 is Healthy Subjects and Group 2 is Focal Segmental Glomerulosclerosis (FSGS).

Gene expression patterns in focal segmental glomerulosclerosis (FSGS) patients were examined using the NephroSeq database. The analysis showed notable similarities in dysregulation linked to chronic kidney injury. Specifically, the ANGPTL4 gene was significantly underexpressed, while the ACTA4 gene was markedly overexpressed according to NephroSeq data. Additionally, RNA sequencing results revealed that treating mesangial cells with MDB-52a effectively normalized these gene expression levels. MDB-52a elevated ANGPTL4 expression to near-normal levels and significantly reduced ACTA4 overexpression. These results indicate that MDB-52a can reduce gene dysregulation and promote recovery of key pathways involved in kidney function. This emphasizes the potential of MDB-52a to reverse molecular changes associated with chronic kidney injury (Figure 9).

## 4. Discussion

Targeted therapies have profoundly changed cancer treatment in recent years. VEGF-TKIs are among the most prominent options in this therapeutic area. These powerful chemotherapeutic agents have attracted significant interest, providing hope to patients fighting cancer. However, despite their effectiveness, VEGF-TKIs like sorafenib are linked to notable side effects, especially related to heart and kidney health. Recent research from our group and others highlights these issues, revealing that VEGF-TKIs can lead to hypertension, proteinuria, and kidney damage [24,25,26]. In a recent study, we found that sorafenib increased renal levels of soluble epoxide hydrolase (sEH), an enzyme crucial for metabolizing epoxyecosatrienoic acid (EET). We also demonstrated that boosting renal EET levels reduced kidney injury in rats treated with sorafenib [27].

In this study, we examined the protective effects of several new 8,9-EET analogs in an in vitro model of sorafenib-induced glomerular damage, using glomerular mesangial cells and podocytes. These cell types were selected because both are vital for glomerular function [28]. Podocytes create the slit diaphragm and are especially susceptible to VEGF-TK inhibition. The slit diaphragm plays a key role in the glomerular filtration barrier, preventing protein from leaking into the urine. Damage to podocytes and this barrier leads to proteinuria, a main indicator of glomerular injury [29]. VEGF-TK inhibition also affects mesangial cells, which support glomerular capillaries; injury here can cause mesangial matrix expansion and glomerulosclerosis. Additionally, the growth of renal mesangial cells and podocytes decreases after a certain point in their lifespan once the glomerulus is fully developed [30].

Our research demonstrates that sorafenib causes significant cytotoxic effects in both glomerular cell types and alters the expression of genes related to podocyte health, pointing to podocytopathy. These results are consistent with numerous preclinical and clinical studies documenting VEGF-TK inhibitor-associated nephrotoxicity, which involves podocyte damage and glomerular mesangiolysis [30]. Several studies also highlight substantial kidney toxicity linked to VEGF-TK inhibition, potentially leading to chronic kidney disease [31]. Moreover, our team previously observed increases in blood pressure, proteinuria, renal intratubular cast formation, interstitial fibrosis, glomerular injury, and nephrin loss in rats treated with sorafenib [27,32]. Both our findings and those from other research emphasize the importance of reducing VEGF-TK inhibitor-induced renal toxicity to better manage cancer patients on therapies affecting VEGF-TK signaling in the kidneys. One promising approach is the use of EET and/or its analogs, which have proven highly effective against various kidney diseases. Their anti-inflammatory, antioxidative, anti-ER stress, anti-apoptotic, and antifibrotic properties are crucial for their kidney-protective effects.

Extensive research has examined analogs of 11,12-EET and 14,15-EET, two of the four EET regioisomers, highlighting their roles in kidney and cardiovascular functions. The biological effects of EETs and their analogs vary across different tissues. Specifically, 8,9-EET analogs are effective against glomerular diseases, unlike 11,12- and 14,15-EET analogs, which do not target the glomerulus to reduce permeability [9,16]. Studies show that 8,9-EET and its analogs safeguard the glomerular filtration barrier from damage caused by FSGS permeability factors [16]. These findings emphasize the specificity of 8,9-EET in protecting kidney structures and point to its potential as a treatment for glomerular diseases. Consequently, we designed a series of novel 8,9-EET analogs featuring an oxamide group. These compounds degrade more slowly, prolonging their activity in the body, and their water-soluble metabolites promote easy excretion via urine, decreasing toxicity risks. Unlike epoxides, which yield diols that can build up and cause toxicity, oxamide-based molecules produce acidic, more-soluble metabolites that are eliminated more readily. We tested twenty of these new oxamide 8,9-EET analogs, observing six (MDB-52a, MDB-52b, MDB-77, MDB-78, RM-84, MDB-32) with notable protective effects on glomerular mesangial cells and podocytes exposed to sorafenib. Among them, MDB-52a was the most effective. Further in vitro studies examined the molecular basis of the cytoprotective effect of the 8,9-EET analogs. First, differential RNA sequencing revealed how sorafenib influences gene expression in HRMCs, showing significant changes in 1244 genes, which indicates a broad impact on cellular functions, particularly those linked to cell cycle and survival pathways.

The observed downregulation of genes encoding key cell cycle regulators, including CDC25, E2F2, MCM10, and MYBL2, suggests a mechanism through which sorafenib inhibits cell proliferation. CDC25, especially CDC25A, plays a vital role in progressing the cell cycle by removing inhibitory phosphates from tyrosine and threonine residues on CDK2, thereby activating the cyclin E-CDK2 and cyclin A-CDK2 complexes, which are essential for the G1 to S phase transition [33]. This activation begins with phosphorylation events during late G1, mainly mediated by cyclin E-CDK2 complexes. Similarly, E2F2 is crucial for transcribing genes necessary for S-phase entry, and its downregulation has been linked to decreased proliferation and increased apoptosis in various cancer models [34]. Based on current findings, we suggest that sorafenib’s inhibition of the genes encoding these proteins contributes to its observed antiproliferative effects, making it a strong inhibitor of cell division. Conversely, the upregulation of genes encoding ANKK1, DUSP9, CYP27B1, and RRAGD indicates complex cellular adaptation to sorafenib treatment. DUSP9 and RRAGD regulate the Raf/MEK/ERK signaling pathway, which is critical for cell survival and proliferation [35]. Sorafenib’s primary mechanism involves targeting the Raf kinase to inhibit this pathway and suppress tumor growth; however, the increased expression of genes encoding DUSP9, a negative regulator of the MAPK pathway, could represent a feedback loop aimed at restoring ERK signaling, a common response in cells attempting to counteract kinase inhibitor effects.

The increased expression of the ANKK1 gene indicates it may enhance HRMCs’ sensitivity to sorafenib, consistent with other cancer models where higher ANKK1 gene and protein levels are linked to improved drug responses [36]. The significance of this upregulation lies in its potential to target compensatory pathways, such as the Raf/MEK/ERK cascade, to boost sorafenib’s effectiveness to kill cells and prevent resistance. These results show that sorafenib has a dual effect on HRMCs: it inhibits proliferation but also activates survival pathways. Comparing HRMCs treated with sorafenib alone versus with MDB-52a shows a notable increase in RP11-178L8.4 with sorafenib, which decreases when MDB-52a is added, suggesting that 10 µM MDB-52a modulates this gene and could shift the molecular environment toward a more glomerular protective state. Several other genes—including ACTA-2, SSC-5D, RP5-977B1.11, and ACTA2-AS1—are significantly downregulated with the combined treatment compared to sorafenib alone. ACTA-2 encodes smooth muscle actin, essential for muscle contraction and tissue integrity, and its reduction is relevant because it is involved in fibrosis, vascular disorders, and cancer, where cytoskeletal dynamics and cell mobility are key. The downregulation of ACTA-2-AS1, an antisense transcript, suggests tightly regulated gene expression, as it influences ACTA-2 through transcriptional interference and chromatin remodeling. Studies show that ACTA-2-AS1 impacts cell migration, adhesion, and extracellular matrix production, vital in fibrosis and tumor metastasis [37,38]. Data from Nephroseq in FSGS patients reveals an opposite pattern with ACTA2 upregulated and ANGPTL4 downregulated, indicating fibrosis and structural damage. The ability of MDB-52a to reverse these gene expression changes, based on RNA-seq, suggests it could restore normal cell functions, support mesangial cell health, and preserve glomerular structure. These findings highlight the potential of oxamide 8,9-EET analogs like MDB-52a to combat fibrosis and support glomerular stability.

Comparing sorafenib alone with the combination at 3 µM MDB-52a shows only one gene, RP11-244H3.4, with significant change, indicating that lower MDB-52a doses have limited effects. The more substantial molecular effects at 10 µM MDB-52a, affecting five genes, demonstrate dose-dependent influence, which could enhance combined therapy effectiveness. The ability of MDB-52a, an 8,9-EET analog, to activate pathways aligns with other studies on EETs’ roles in inflammation, apoptosis, and angiogenesis [9]. This study found MDB-52a has a strong protective effect against sorafenib-induced nephrotoxicity, maintaining podocyte gene expression. Sorafenib harms the kidneys by decreasing critical podocyte markers such as NPHS1, NPHS2, Synaptopodin, CD2AP, ITGB, and TJP1. Disruption of NPHS1 and NPHS2 impairs slit diaphragm function, and reduced synaptopodin increases podocyte vulnerability [39,40]. These changes compromise podocyte structure and function, essential for glomerular filtration and kidney health. MDB-52a, at higher doses, shows a dose-dependent protective effect by restoring podocyte markers like NPHS1, synaptopodin, and TJP1, while lowering desmin, an injury marker. Synaptopodin supports podocyte stability by maintaining the cytoskeleton, and its increase suggests improved resilience, while restored TJP1 helps preserve the glomerular barrier. The decrease in desmin further indicates protection against cytoskeletal damage. These gene expression changes point to molecular targets for MDB-52a’s protective effects, emphasizing its potential to mitigate sorafenib-induced nephrotoxicity. Restoring key podocyte and mesangial cell genes, such as ANGPTL4 and ACTA4, underscores MDB-52a’s promise in reversing molecular changes linked to chronic kidney injury.

## 5. Conclusions

Our results highlight the therapeutic potential of 8,9-EET analogs in glomerular disease, as they preserve glomerular cells and lessen renal toxicity, opening avenues for further research into their clinical use to improve kidney safety during cancer treatments. Most notably, we have identified a promising lead, the oxamide 8,9-EET analog MDB-52a, for additional in vivo studies. However, it is important to recognize that although this research offers valuable transcriptomic insights, a key limitation is the lack of protein-level validation of the genes significantly affected by sorafenib and MDB-52a. Future work should include techniques like Western blotting, ELISA, or immunofluorescence to verify whether gene expression changes correspond to alterations in protein levels and activity. Overcoming this limitation will deepen our understanding of the therapeutic mechanisms and confirm MDB-52a’s potential as a protective agent against VEGF-TKI-induced kidney toxicity.

## Figures and Tables

**Table 1:** List of compounds used in this study.

Serial No.	Compound ID	Compound Structure	Molecular Weight
1	MDB-31	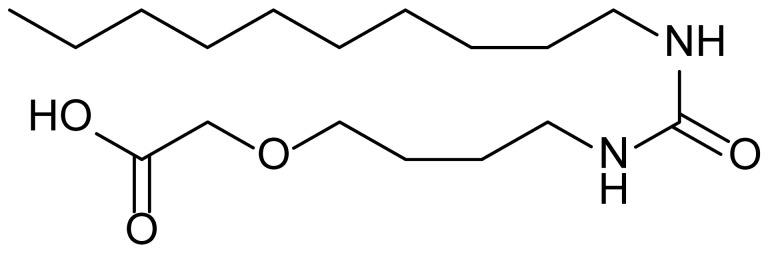	330.5
2	MDB-32	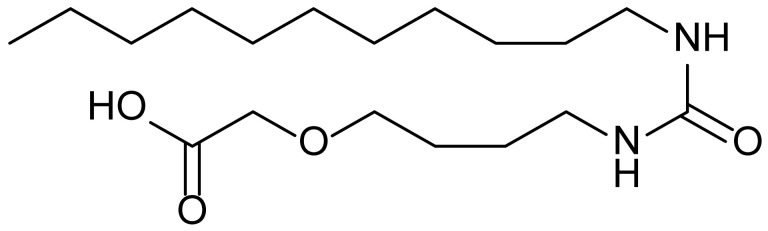	344.5
3	MDB-33	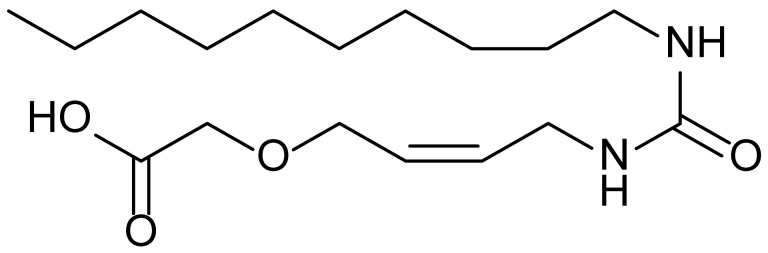	328.5
4	MDB-41	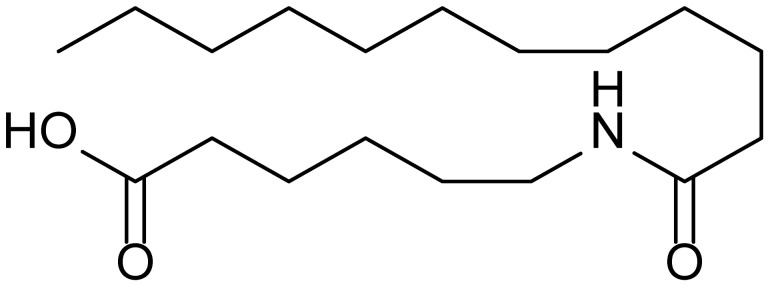	313.5
5	MDB-44	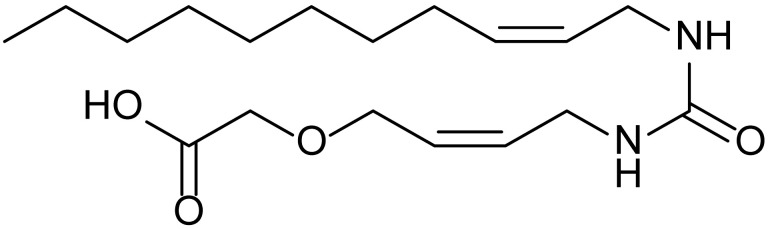	340.5
6	MDB-46	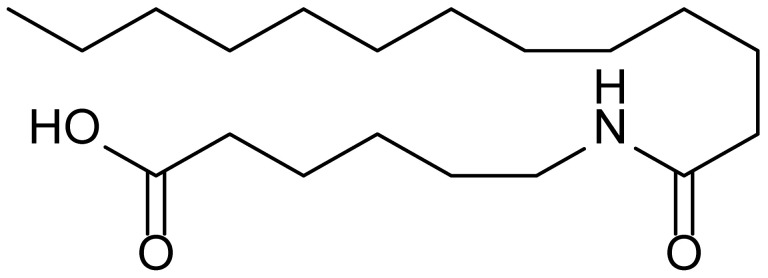	327.5
7	MDB-52a	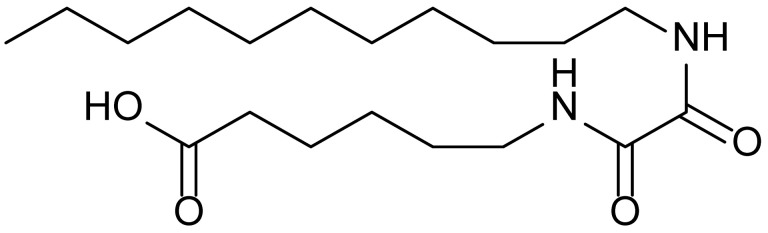	365.5
8	MDB-52b	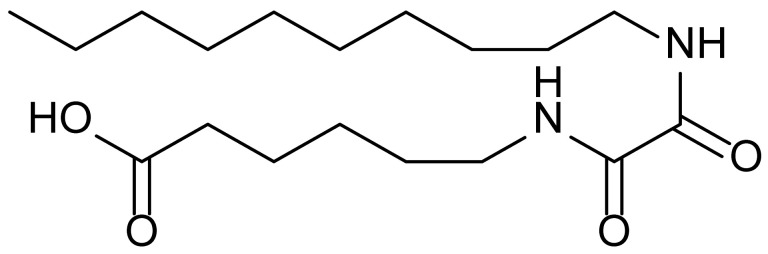	342.5
9	MDB-58	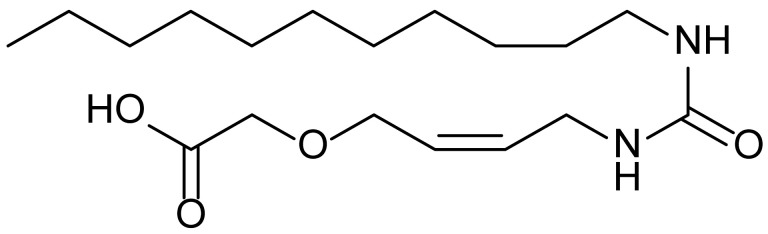	342.5
10	MDB-76	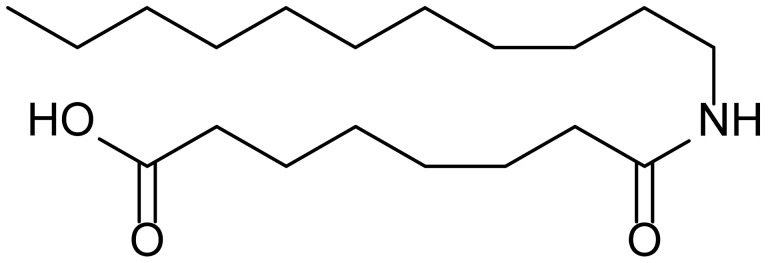	327.5
11	MDB-77	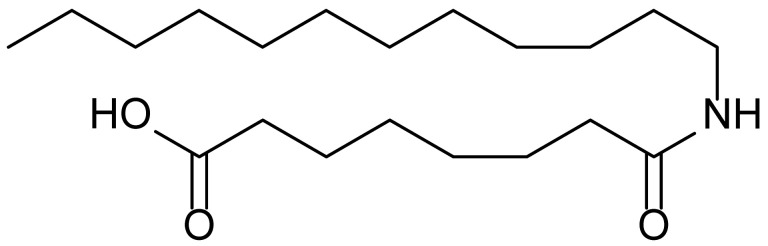	341.5
12	MDB-78	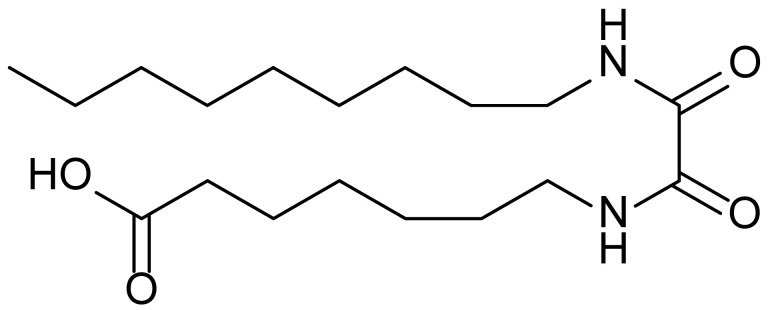	342.5
13	MDB-79	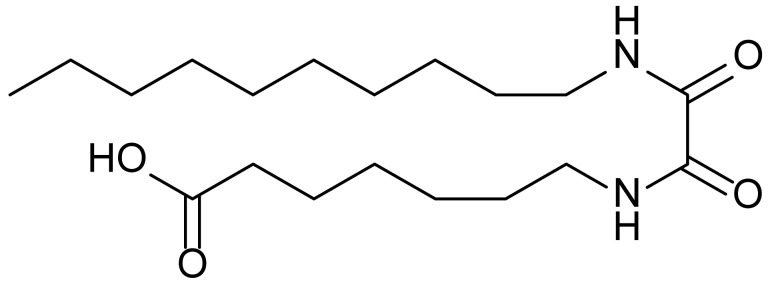	356.5
14	MDB-80	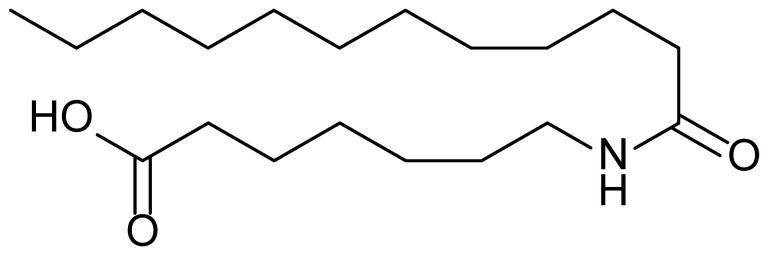	327.5
15	MDB-81	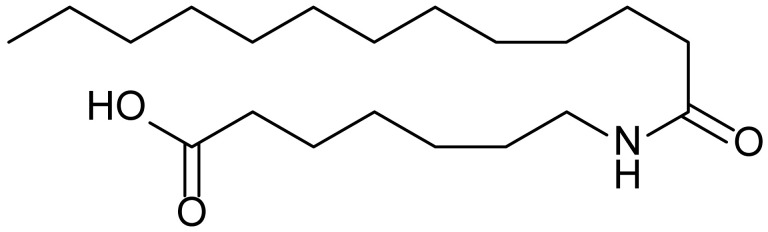	341.5
16	MDB-18	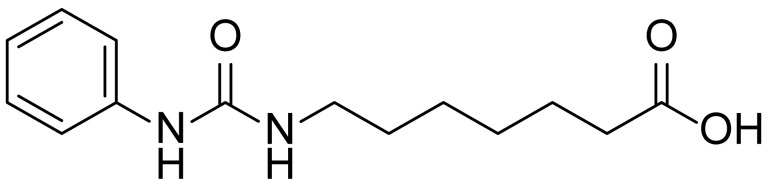	328.5
17	RM-69	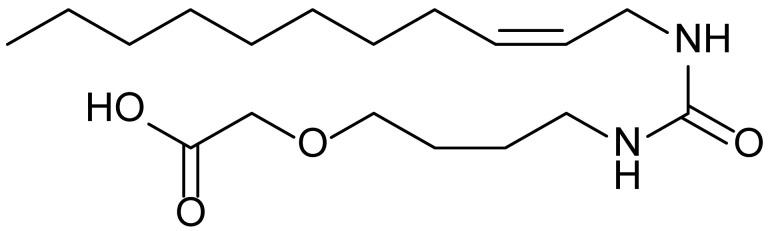	342.5
18	RM-72	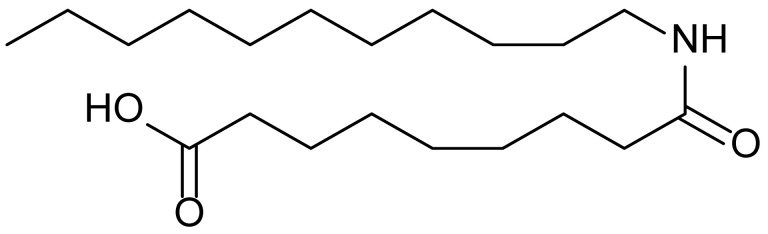	341.5
19	RM-81	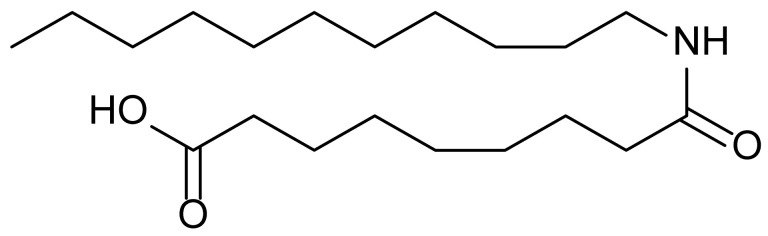	339.5
20	RM-84	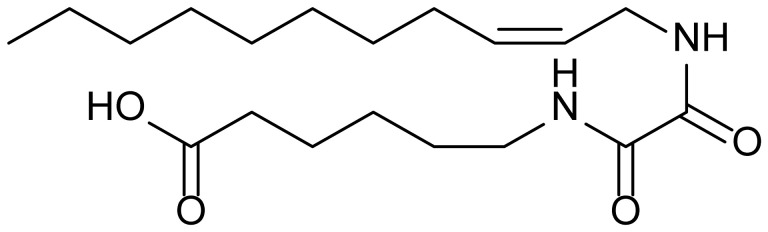	354.5

## Data Availability

The raw data supporting the conclusion of this article will be made available by the authors without undue reservation.

## Supplementary Materials

The following supporting information can be downloaded at https://www.mdpi.com/article/10.3390/biom15091324/s1: Figure S1: Effects of 8,9-EET analogs on WST activity for cell viability in HRMC Cells; Figure S2: Effects of 8,9-EET analogs on WST activity for cell viability in HRMC Cells; Figure S3: Effects of 8,9-EET analogs on WST activity for cell viability in podocyte cells; Figure S4: Evaluation of the effects of various 8,9-EET analogs on HRMC cell confluence under sorafenib treatment; Figure S5: Evaluation of the effects of various 8,9-EET analogs on podocyte cell confluence under sorafenib treatment; Table S1: List of Primer used in this study for RT-qPCR; Table S2: Group B vs. Group A; Table S3: Group D vs. Group B; Table S4: Group E vs. Group A.
