# Supplementary material for: EET-Based Therapeutics Mitigate Sorafenib-Associated Glomerular Cell Damage
Source: Biomolecules. 2025 Sep 16;15(9):1324. doi: 10.3390/biom15091324 (PMC12467510; doi:10.3390/biom15091324)
Supplement: Supplementary file 1 [file biomolecules-15-01324-s001.zip › biomolecules-3671812-Supplementary-revision v3.docx]

**SUPPLEMENTARY**

**

**

**Supplementary Figure 1: Effects of 8,9-EET analogs on WST activity for cell viability in HRMC Cells.**

We evaluated the effects of 8,9-EET analogs and sorafenib on the viability of human renal HRMC cells using the WST assay. Five treatments were assessed: Control (no treatment), Sorafenib (5 µM) as a vehicle, and Sorafenib (5 µM) combined with 8,9-EET analogs at concentrations of 1 µM, 3 µM, and 10 µM. This assay measured the cell viability under these conditions to assess the protective effects of 8,9-EET analogs against sorafenib-induced cytotoxicity.

**
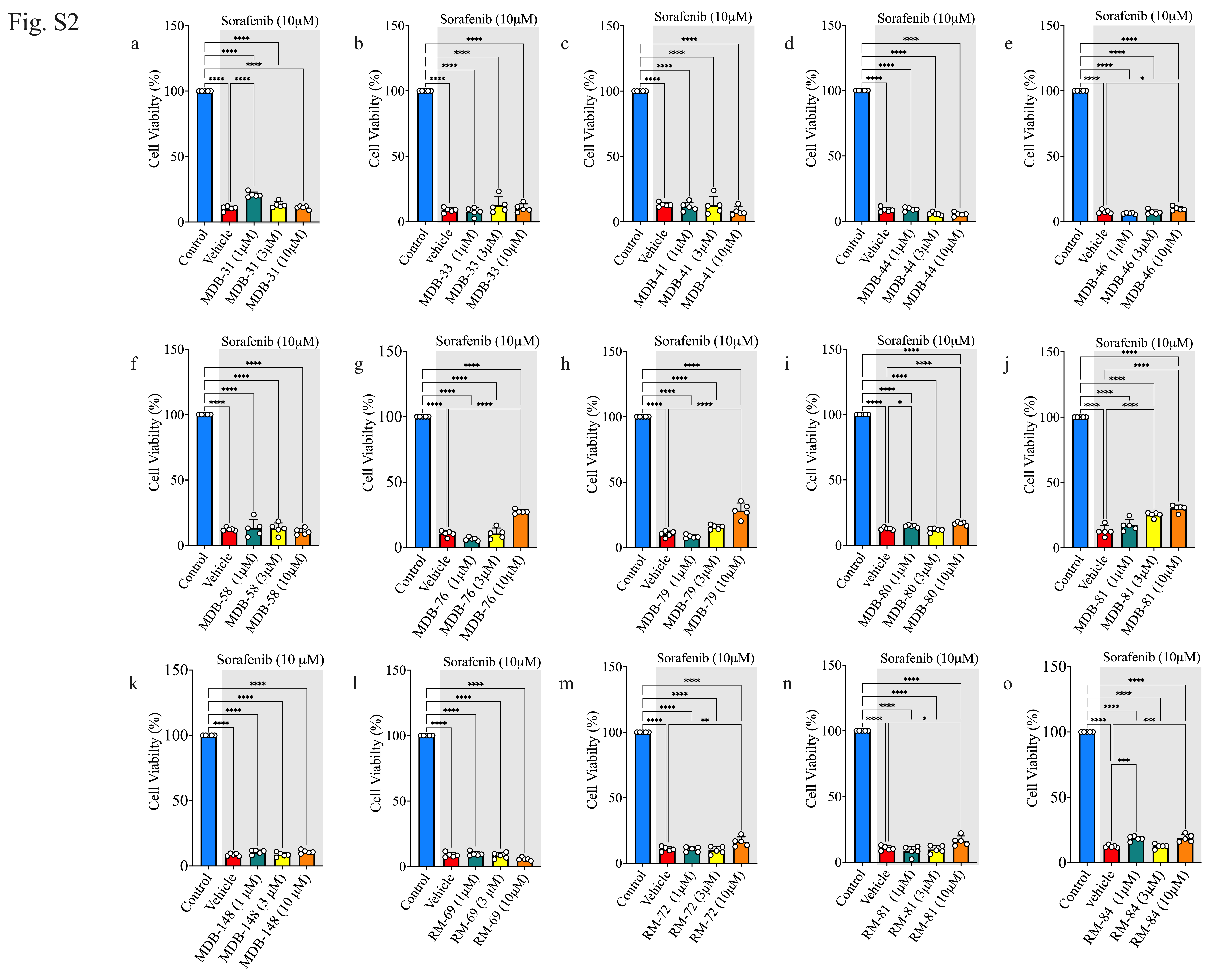
**

**Supplementary Figure 2: Effects of 8,9-EET analogs on WST activity for cell viability in HRMC Cells.**

We evaluated the effects of 8,9-EET analogs and sorafenib on the viability of human renal HRMC cells using the WST assay. Five treatments were assessed: Control (no treatment), Sorafenib (10 µM) as a vehicle, and Sorafenib (10 µM) combined with 8,9-EET analogs at concentrations of 1 µM, 3 µM, and 10 µM. This assay aimed to determine the protective effects of the 8,9-EET analogs against the cytotoxicity induced by higher concentrations of sorafenib.


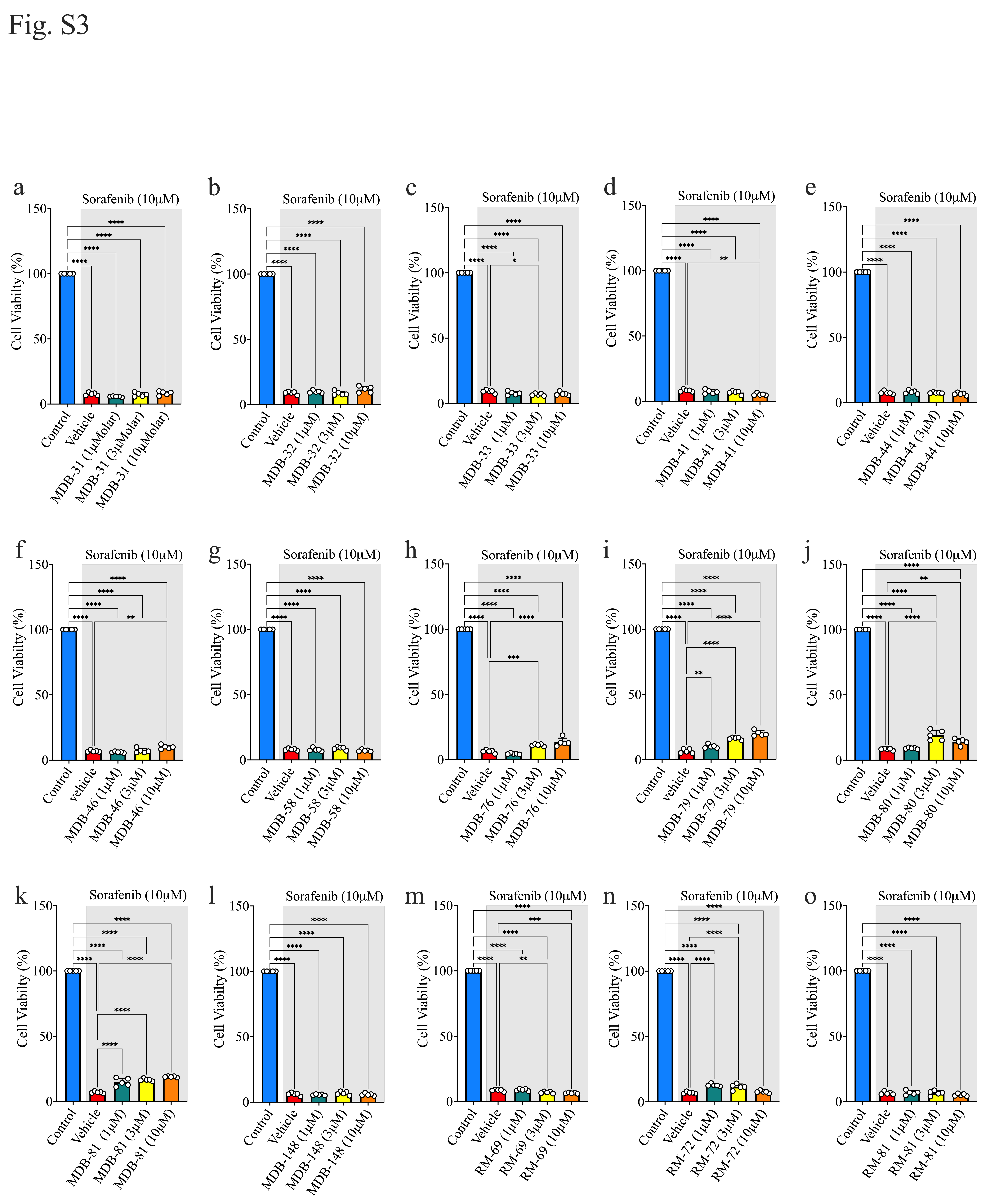


**Supplementary Figure 3: Effects of 8,9-EET analogs on WST activity for cell viability in podocyte cells.**

We assessed the impact of 8,9-EET analogs and sorafenib on the viability of human renal podocyte cells using the WST assay. Five treatment groups were evaluated: Control (no treatment), Sorafenib (10 µM) as a vehicle, and Sorafenib (10 µM) combined with 8,9-EET analogs at concentrations of 1 µM, 3 µM, and 10 µM. This experiment aimed to determine the protective effects of 8,9-EET analogs against sorafenib-induced cytotoxicity in podocyte cells.


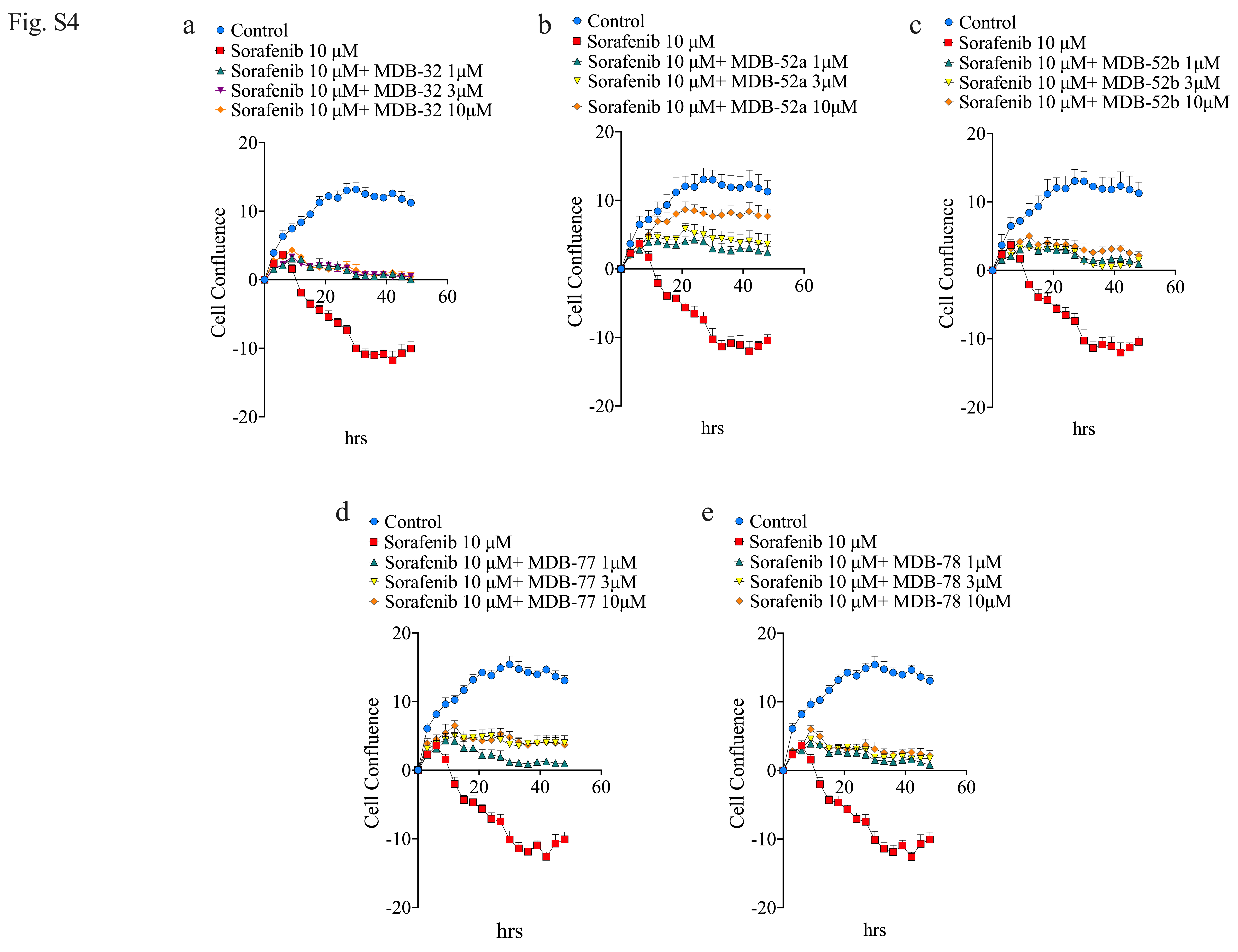


**Supplementary Figure 4: Evaluation of the effects of various 8,9-EET analogs on HRMC cell confluence under sorafenib treatment.**

(a) HRMCs treated with 10 µM sorafenib exhibit a rapid decrease in cell confluence, indicative of significant cytotoxicity. In contrast, cells co-treated with MDB-32 show improved cell confluence compared to sorafenib-only treatment, suggesting protective effects against sorafenib-induced cell death. (b) MDB-52a demonstrates substantial restoration of cell confluence across all tested concentrations (1, 3, and 10 µM), with the 10 µM dose nearly restoring confluence to levels comparable to control cells. (c-e) MDB-52b, MDB-77, and MDB-78 display dose-dependent restoration of cell confluence, with higher concentrations providing increased protection against sorafenib-induced cytotoxic effects. These findings underscore the potential of 8,9-EET analogs, particularly MDB-52a, in mitigating sorafenib-induced cytotoxicity and promoting HRMC survival, with notable efficacy at higher concentrations.


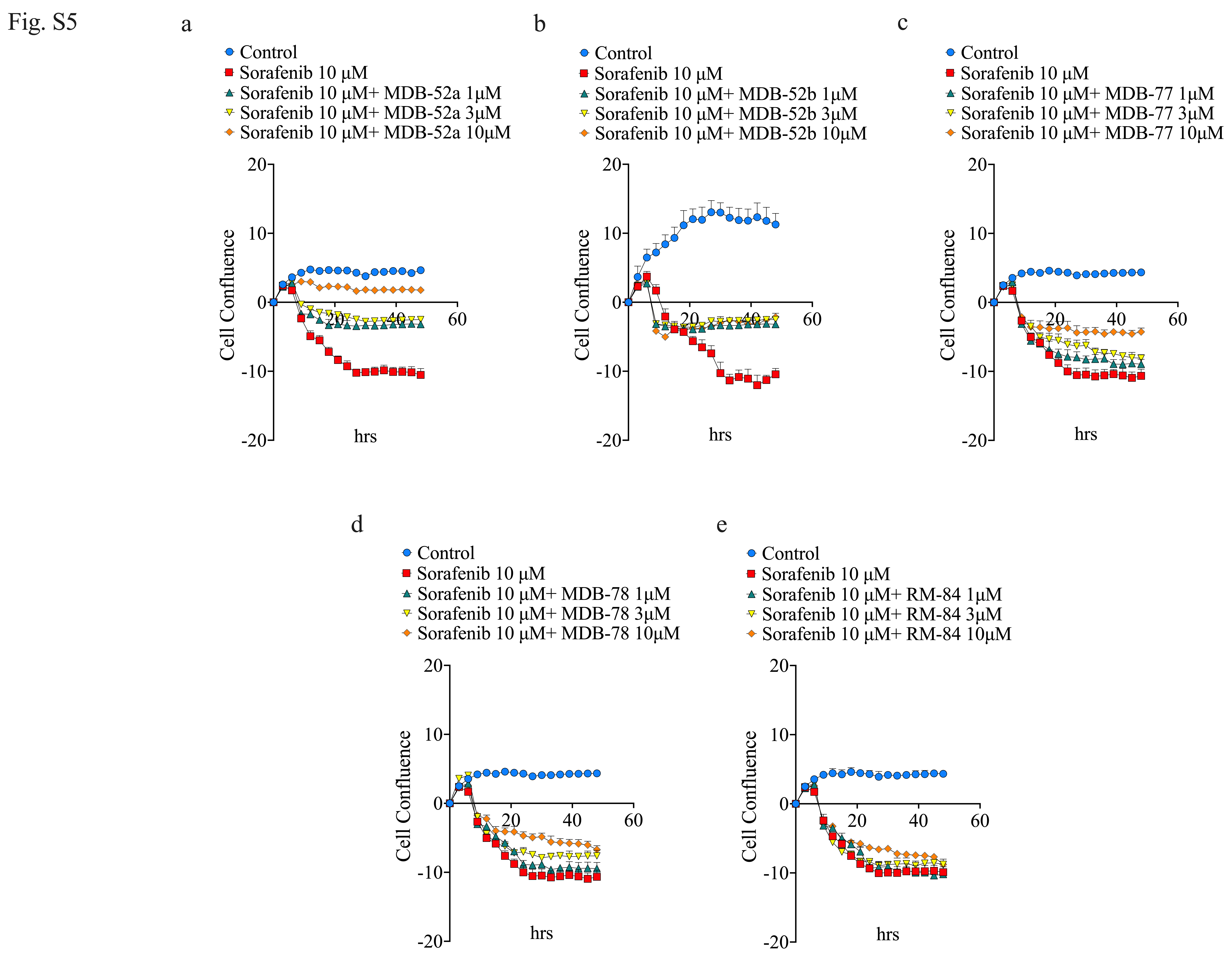


**Supplementary Figure 5: Evaluation of the effects of various 8,9-EET analogs on podocyte cell confluence under sorafenib treatment.**

(a) MDB-52a significantly preserves cell confluence in podocytes, providing protection against sorafenib-induced cytotoxicity. This effect is observed across all tested concentrations (1, 3, and 10 µM), with the 10 µM dose showing the most notable improvement, while 10 µM sorafenib alone results in a marked reduction in cell confluence. (b-e) MDB-52b, MDB-77, MDB-78, and RM-84 demonstrate a dose-dependent preservation of cell confluence, with higher concentrations providing greater protection against sorafenib-induced cytotoxic effects. These findings highlight the therapeutic potential of 8,9-EET analogs in mitigating sorafenib-induced cytotoxicity and promoting podocyte survival, with MDB-52a exhibiting the most pronounced protective effect at 10 µM.

**Supplementary Table 1:** List of Primer used in this study for RT-qPCR.

| Gene Name |  |  |
| --- | --- | --- |
| ACTB | Forward Primer | CACAGAGCCTCGCCTTTGC |
|  | Reverse Primer | GCGCGGCGATATCATCATCC |
| NPHS1 | Forward Primer | GTCTGCACTGTCGATGCCAATC |
|  | Reverse Primer | CCAGTTTGGCATGGTGAATCCG |
| Desmin | Forward Primer | TCCAGTCCTACACCTGCGAGAT |
|  | Reverse Primer | CGCAATGTTGTCCTGGTAGCCA |
| Synpo | Forward Primer | AGGAGGTGAGATGCAGCACACT |
|  | Reverse Primer | TAGGGTGTTGGGCTGGATGTCA |
| NPHS2 | Forward Primer | CTGTGAGTGGCTTCTTGTCCTC |
|  | Reverse Primer | CCTTTGGCTCTTCCAGGAAGCA |
| CD2AP | Forward Primer | CCAAAGCCTGAACTGATAGCTGC |
|  | Reverse Primer | GGACTTGTGGAGCTGCTGGTTT |
| ITGB1 | Forward Primer | GGATTCTCCAGAAGGTGGTTTCG |
|  | Reverse Primer | TGCCACCAAGTTTCCCATCTCC |
| TJP1 | Forward Primer | GTCCAGAATCTCGGAAAAGTGCC |
|  | Reverse Primer | CTTTCAGCGCACCATACCAACC |
